# Celecoxib reverses the glioblastoma chemo-resistance to temozolomide through mitochondrial metabolism

**DOI:** 10.18632/aging.203443

**Published:** 2021-09-08

**Authors:** Delong Yin, Guoqing Jin, Hong He, Wei Zhou, Zhenbo Fan, Chen Gong, Jing Zhao, Huihua Xiong

**Affiliations:** 1 Department of Orthopaedics, The Third Affiliated Hospital of Guangzhou Medical University, Guangzhou 510150, China; 2 Department of Intensive Care Unit, The Third Affiliated Hospital of Guangzhou Medical University, Guangzhou 510150, China; 3 Department of Obstetrics and Gynecology, Key Laboratory for Major Obstetric Diseases of Guangdong Province, The Third Affiliated Hospital of Guangzhou Medical University, Guangzhou 510150, China; 4 Department of Oncology, Tongji Hospital, Tongji Medical College, Huazhong University of Science and Technology, Wuhan 430030, China

**Keywords:** glioblastoma, chemo-resistance, temozolomide, mitochondrial metabolism, cyclooxygenase-2

## Abstract

Temozolomide (TMZ) is used for the treatment of high-grade gliomas. Acquired chemoresistance is a serious limitation to the therapy with more than 90% of recurrent gliomas showing little response to a second line of chemotherapy. Therefore, it is necessary to explore an alternative strategy to enhance the sensitivity of glioblastoma (GBM) to TMZ in neuro-oncology. Celecoxib is well known and widely used in anti-inflammatory and analgesic. Cyclooxygenase-2 (COX-2) expression has been linked to the prognosis, angiogenesis, and radiation sensitivity of many malignancies such as primitive neuroectodermal tumor and advanced melanoma. The objective of this study was to explore the chemotherapy-sensitizing effect of celecoxib on TMZ in GBM cells and its potential mechanisms. From the study, we found that the combination therapy (TMZ 250uM+celecoxib 30uM) showed excellent inhibitory effect to the GBM, the LN229 and LN18, which were the TMZ resistant GBM cell lines. Our data suggest that the combination therapy may inhibits cell proliferation, increases apoptosis, and increases the autophagy on LN229 and LN18. The potential molecular mechanisms were related to mitochondrial metabolism and respiratory chain inhibition.

## INTRODUCTION

GBM is an aggressive and prevalent brain tumor of the astrocytic lineage characterized as a high-grade tumor of the central nervous system [1]. The median overall survival for patients with GBM is between 12 to 15 months. The standard treatment for GBM consists of tumor removal followed by radiotherapy with concurrent and adjuvant TMZ [2]. Although TMZ is commonly used in the adjunctive treatment of gliomas and can efficiently inhibit proliferation and induce apoptosis of GBM cells, the prognosis of GBM remains poor [3]. Chemotherapy becomes impaired by development of chemo-resistance, especially for the patients with GBM who are frequently exhibited an early deterioration of performance status [4], with the 5-year survival rate of just 9% [5]. This phenomenon presents the most challenging barrier in the successful treatment of GBM and is the principal reason for chemotherapy failure [2, 6].

GBM chemo-resistance includes congenital and acquired resistance, and the acquired chemo-resistance is more serious. More than 90% of patients with recurrent gliomas have acquired chemo-resistance showing little response to a second line of chemotherapy [7]. Therefore, it is urgently needed to find a strategy to enhance the sensitivity of TMZ in the treatment of GBM patients.

Cyclooxygenase-2 (COX-2) expression has been linked to the prognosis, angiogenesis and radiation sensitivity of many malignancies. Joki et al. [8] have reported that treatment with a COX-2 inhibitor, NS-398, reduced tumor cell migration and proliferation and increased apoptosis in mice bearing xenografts of U-87MG and U-251MG glioma cell lines. And COX-2 protein has been noted in greater amounts in high-grade gliomas than in low-grade gliomas or normal brain, it also shows a link of poorer survival in patients with malignant gliomas that have increased COX-2 expression [9]. Celecoxib is a selected COX-2 inhibitor, which has been evaluated the effect on GBM radiotherapy [10]. Although this study did not get the expected results, it provided important preliminary data and set the stage for future trials evaluating combination therapy with radiation, TMZ, and celecoxib in those patient population. This study focuses on the reversal effect and potential mechanism of celecoxib on TMZ resistance in glioma.

## RESULTS

### Low concentrations of celecoxib did not influence the proliferation of GBM cells but enhanced the anti-proliferation of TMZ

**Figure 1:**
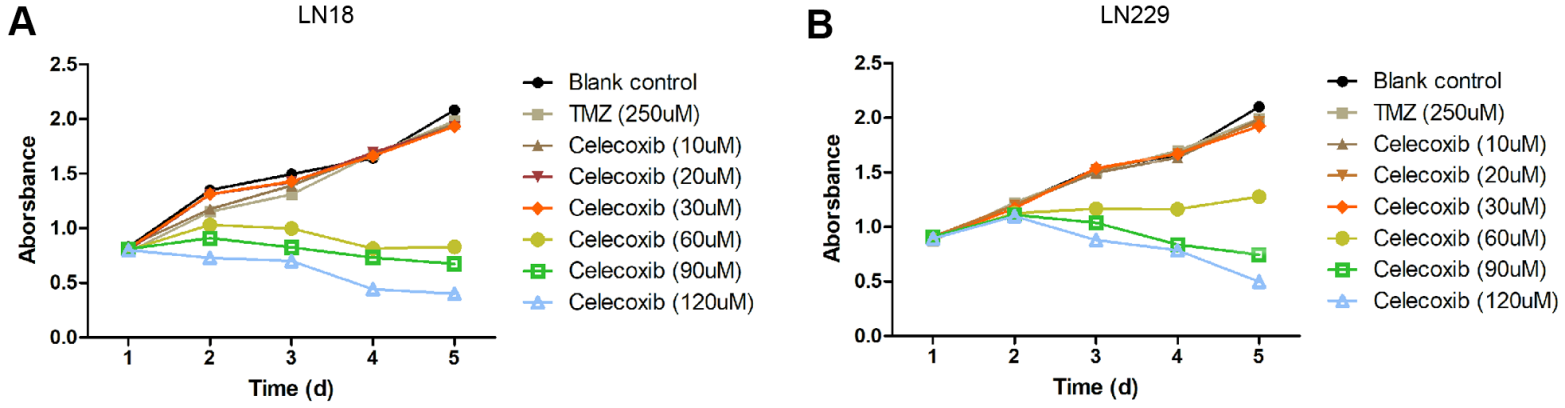
**Cytostatic effects of celecoxib on GBM LN18 and LN229 cells assay by MTS.** (**A**) LN18 cells (5×10^3^) were seeded into 96-well plates, and the absorbance of the cells was detected at day1 to day5 in culture medium under various conditions, as indicated. (**B**) LN229 cells (5×10^3^) were seeded into 96-well plates, and the absorbance of the cells was detected at day1 to day5 in culture medium under various conditions, as indicated. Data shows mean Absorbance 490nm and ±SEM, four independent wells per condition.

**Figure 2:**
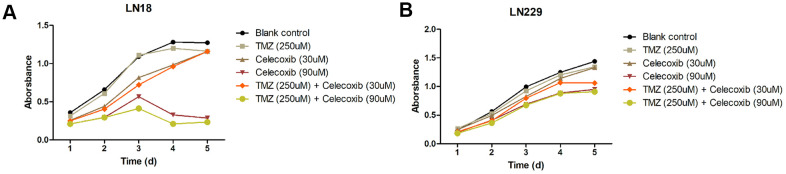
**Cytostatic effects of TMZ and/or celecoxib on LN18 and LN229 GBM cells assay by MTS.** (**A**) LN18 cells (5×10^3^) were seeded into 96-well plates, and the absorbance of the cells was detected at day1 to day5 in culture medium under various conditions, as indicated. (**B**) LN229 cells (5×10^3^) were seeded into 96-well plates, and the absorbance of the cells was detected at day1 to day5 in culture medium under various conditions, as indicated. Data shows mean Absorbance 490nm and ±SEM, four independent wells per condition.

The GBM cell lines, LN229 and LN18, were resistant to TMZ treatment had been reported [11–13]. In our study, we selected these two cell lines to perform cytotoxicity assay with MTS. The LN229 and LN18 cells were treated with celecoxib with 10uM, 20uM, 30uM, 60uM, 90uM, 120uM and TMZ 250uM independently, then we add the MTS to test the cell cytotoxicity / proliferation. The results showed that both cell lines were resistant to 250uM TMZ from D1 to D5, and not significantly inhibited by 30uM of celecoxib. But when the celecoxib dose up to 60uM and more, the LN18 cell line was inhibited at D2. The LN229 cell line was also inhibited at the same dose of celecoxib but was later than LN18, when the LN229 was treated with celecoxib dose up to 60uM and more at D3 (Supplementary Table 1 for specific data), these results were consistent with previous research [14, 15]. Both LN18 and LN229 cell lines were resistant to the TMZ, which were also consistent with the results of previous studies [11, 12], the 60uM and more dose of celecoxib has obvious inhibitory effect on LN18 and LN229 proliferation at D2 or D3 (Figure 1A, 1B and Supplementary Table 1). Then we treated the LN229 and LN18 cells with the TMZ, celecoxib and combination, the MTS assay results showed that the LN229 and LN18 cell line were obviously suppressed with TMZ 250uM+celecoxib 30uM (Figure 2A, 2B and Supplementary Table 2). Yamaguchi et al. [16]. recently reported that celecoxib could play a role in anti-tumor effects in GBM, and our results found that celecoxib may have a chemo-sensitization effect on TMZ. Neither TMZ 250uM nor celecoxib 30uM can restrict cell proliferation, but the combination therapy suppressed the cell proliferation on D2 for LN18 and D3 for LN229, especially in the comparison of TMZ and combination therapy (Supplementary Table 2). Because celecoxib is used as a sensitization drug, rather than a treatment drug, we chose 30uM as the working concentration that was used for subsequent experiments.

### Celecoxib can enhance the anti-clone formation effect of TMZ on GBM

**Figure 3:**
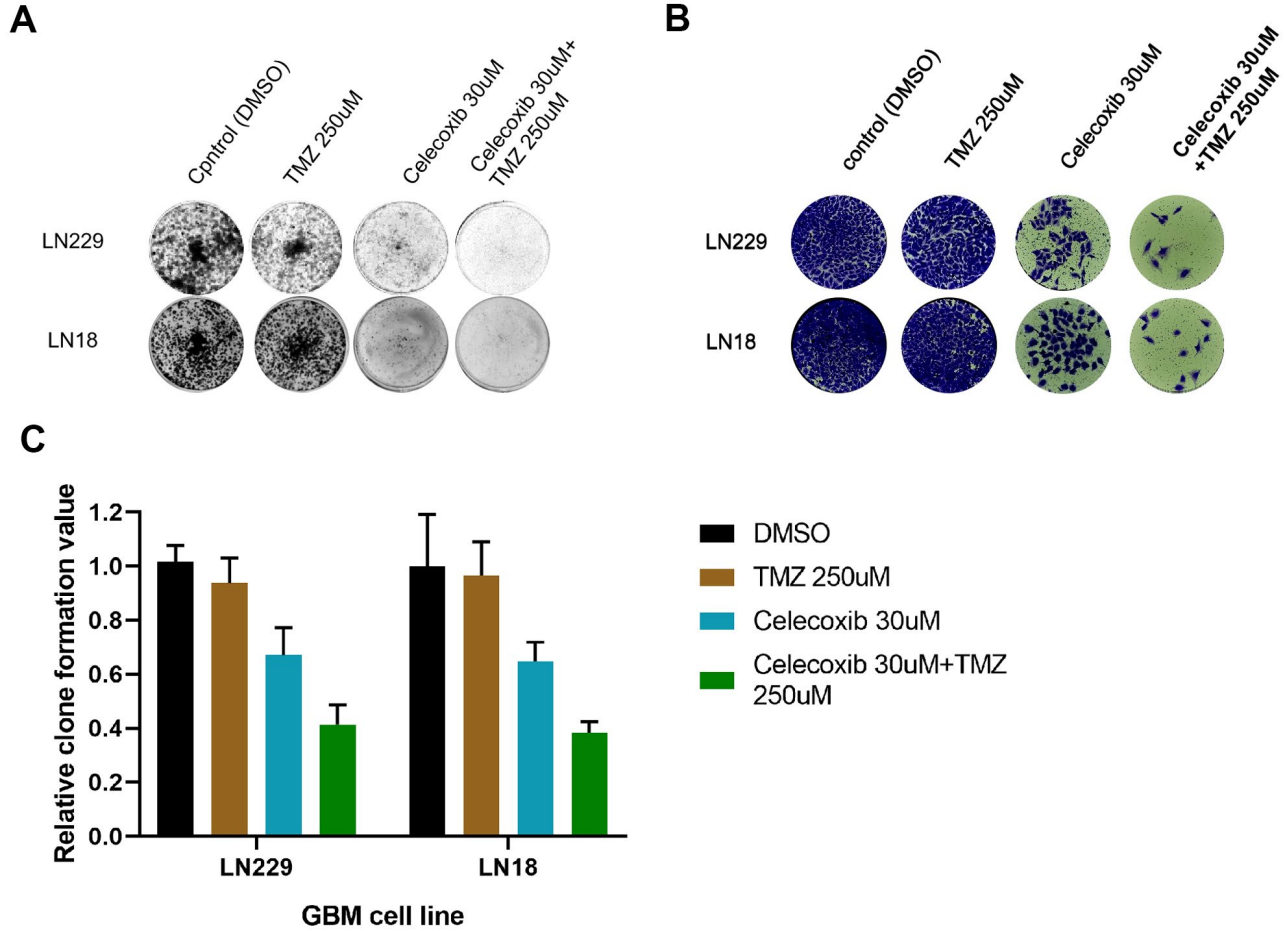
**TMZ and/or celecoxib inhibited colony formation on LN18 and LN229 GBM cells via assay by crystal violet staining.** (**A**) LN18 and LN229 GBM cells (2.5×10^3^) were seeded into 12-well plates, following TMZ and/or celecoxib treatment for 12 days prior to staining. Then the plates were imaged by Bio-Rad equipment. (n=5). (**B**) After staining of LN18 and LN229 GBM cells, the plates were imaged by EVOS Cell Imaging Systems. (**C**) The staining plates were calculated by Image Lab software, and the chart was shown the (A) result. Data shows mean Value and ±SEM (n=5).

The LN229 and LN18 cell lines have been treated with TMZ, celecoxib and combination with 10-14d, then tested by the crystal violet staining (CVS) assay. The results showed LN229, and LN18 cell lines proliferation had been inhabited (Figure 3A, 3B). Especially the combined therapy was more sensitive (Figure 3C, 3D) than single one. The LN229 was more sensitive to the combined therapy (TMZ 250uM+celecoxib 30uM) than LN18 (Figure 3B) clearly from the quantitative analysis (Figure 3C and Supplementary Table 3). This further confirmed the MTS results (Figure 2A, 2B), TMZ-resistant GBM cells were killed by TMZ under the subsidy of celecoxib. And these were basically consistent with previous research [17].

### Celecoxib can enhance the pro-apoptotic effect of TMZ on GBM

**Figure 4:**
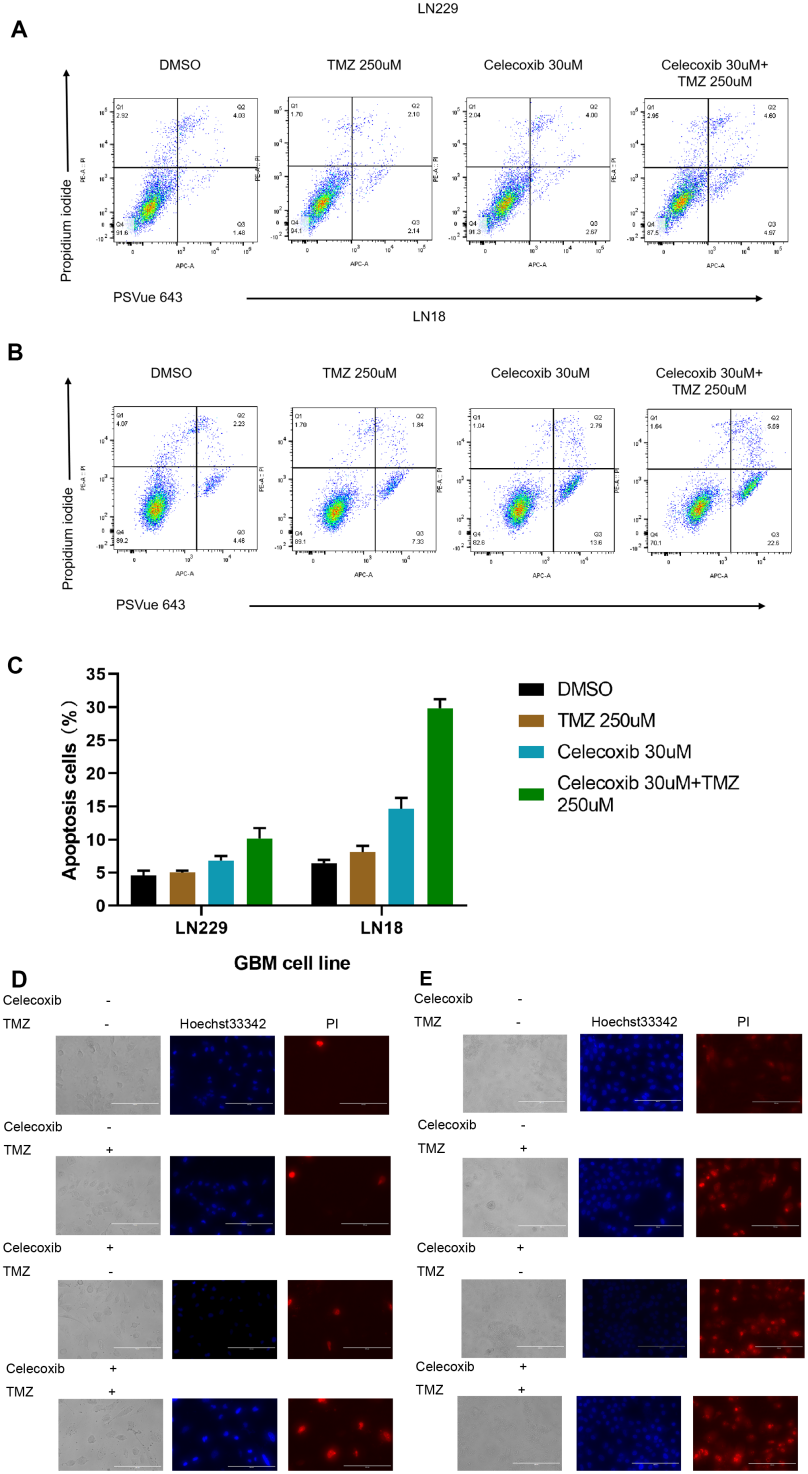
TMZ and/or celecoxib induces cell apoptosis in LN18 and LN229 GBM cells (**A**) the LN229 cells (5×10^3^) were seeded into 12-well plates, (three independent wells per condition) following TMZ and/or celecoxib treatment for 72 hours prior to assay. Then the cells were collected and washed twice with cold PBS and incubated with PI and PSVue 643 at room temperature for 30mins before test by flow cytometer. (**B**) The LN18 cells were practice as same condition. (**C**) The statistical results of the flow cytometry analysis of cell apoptosis. Data shows mean Value and ±SEM (n=5). (**D**) The LN229 cells (5×10^3^) were seeded into 12-well plates, (three independent wells per condition) following TMZ and/or celecoxib treatment for 48 hours prior to assay. Then the cells were collected and washed twice with cold PBS and incubated with PI and Hoechst 33342 at dark room for 5mins then test with Fluorescence microscopy. (**E**) The LN18 cells were practice as same condition (Standard line: 200um).

Apoptosis is an important mechanism of anti-tumor drugs [18]. To address the cellular mechanism of effective for the combination therapy to the GBM. LN229 and LN18 have been treated with TMZ in the presence or absence of celecoxib for 72h and have been stained the cells with PSVue 643 and Propidium Iodide for apoptosis analysis by FACS. The results showed that combination treatment with celecoxib resulted in a significant enhancement of apoptosis in these cell cultures (Figure 4A, 4B and Supplementary Table 4).

To account for cell apoptosis, LN229 and LN18 have been treated with TMZ in the presence or absence of celecoxib for 48h and have been stained the cells with Hoechst 33342 and Propidium Iodide, then analysis with fluorescence microscope. The results showed that the combined treatment significantly enhances cell apoptosis (Figure 4D, 4E). Together, these results showed that GBM cell treated with combined therapy (TMZ 250uM+celecoxib 30uM) has been induced apoptosis significantly.

### The sensitization effect of celecoxib on TMZ might be via OXPHOS and mitobiogenesis

**Figure 5:**
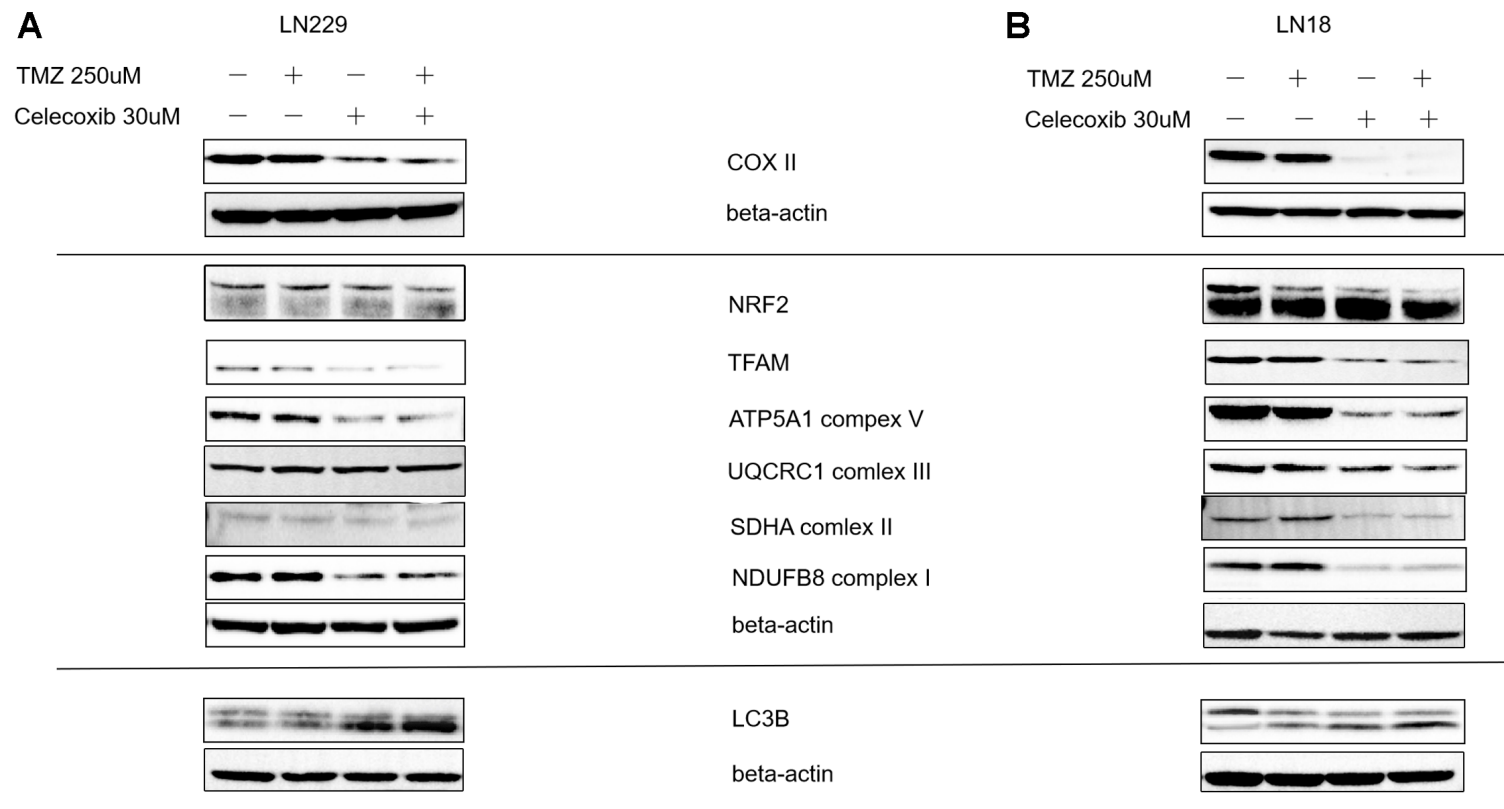
**Regulator of MitoBiogenesis proteins and OXPHOS Subunits were modulated by celecoxib in GBM LN229 and LN18 cells.** (**A**, **B**) LN229 and LN18 cells (2×10^5^) were seeded into 6-well plates, (three independent wells per condition) following TMZ and/or celecoxib treatment for 48 hours prior to assay. Then the lysates harvested were analyzed by Western blotting for various proteins using Beta-Actin as a loading control as indicated. The COX-II was down-regulated by Celecoxib. The OXPHOS Subunits such as complex I, II, V were down-regulated by celecoxib compare the Beta-actin as standard. And the TFAM which was the regulator MitoBiogenesis protein also was down regulated in LN229. The MitoBiogenesis proteins NRF2 was downregulated by celecoxib, combination therapy was more significant. The LC3 protein tends to increase when the LN229 and LN18 were treated with celecoxib and combination.

To find the underlying molecular mechanism of how celecoxib enhances the TMZ inhibited the GBM, we treated the LN229 and LN18 with TMZ in the presence or absence of celecoxib for 72h and collected the proteins, then did the western blot (Wb) assay of oxidative phosphorylation (OXPHOS) and mitobiogenesis proteins. First, these two cell lines showed the down regulated the COX-2 protein during being treated with the celecoxib regardless of the existence of TMZ (Figure 5A). Second, the OXPHOS protein changes were observed, which were represented as complexes I, II, V down regulated during being treated with the combination therapy (Figure 5B). Finally, Mitobiogenesis protein changed in GBM cell lines, which were represented as NRF2 down regulated and LC3B up regulated during treated with the combination therapy (Figure 5C).

Oliva et al. [7]. reported that TMZ-mediated alterations in mitochondrial DNA (mtDNA) and respiratory function contribute to TMZ-dependent acquired chemoresistance. In our study, the results showed that control group respiratory complexes I, II, V were high express, in contrast, they were down regulated when the LN229 and LN18 were treated with 30uM celecoxib in the presence or absence of TMZ. The COX-2 was also a part of the complexes IV [19], and it was down regulated when the LN229 and LN18 treated with celecoxib with or without the TMZ, show that the part of complexes IV was also destroyed. Collectively, similar to previous research points [7], our results suggested that celecoxib could make the resistant GBM cells regain sensitivity to TMZ by impact the electron transport chain (ETC). Previous study showed that TMZ induced profound changes in the activities of the mitochondrial ETC and cellular bioenergetic function [7]. OXPHOS has a central role in cellular energy, the OXPHOS electron transport chain (ETC) comprises four complexes (I to IV) that transfer electrons from donors generated by the TCA cycle and fatty acid oxidation to oxygen, Complex V (F0F1 ATP synthase) uses the stored energy in the proton gradient to generate ATP. Shi et al. [20] also reported that inhibits the activity of F0F1ATP synthase would specifically inhibit the growth of high-throughput GBM sphere cells. Our results showed that celecoxib inhibited the complexes V and increased sensitivity of TMZ to resistant GBM cells consistent with recently study [20].

Mitochondrial transcription factor A (TFAM) is a protein that maintains mtDNA integrity [21]. In GBM, the TFAM RNA and protein levels are upregulated, compared to non-neoplastic brain tissue [22], and the protein levels of TFAM are positively correlated with the malignancy of GBM [23]. In our study, the TFAM has been down regulated in LN229 and LN18 by celecoxib and combination therapy (Figure 5B). This suggested that the combination and celecoxib influenced the TFAM and down regulated the TFAM and consistent with the previous results, the complexes I, II, V (Figure 5B).

In our study, the LN229 and LN18 treated with celecoxib in the presence of TMZ shown the significantly upregulated of LC3B (Figure 5C), consistent with previous research that celecoxib induced marked autophagy [15], particularly in hypoxic cells [14]. And previous research has proven that highly aggressive brain tumors characterized by profound hypoxia, especially in the GBM [24, 25]. In our study, the p62 is upregulate while the LC3B upregulated at the protein levels in the LN229 cells when treated with celecoxib and TMZ (Figure 5C). This is consistent with previous study, that is both p62 and LC3 proteins are highly expressed in mouse tumor implantation model when the tumor is reduced [26]. We also observed the NRF2 was down regulated on LN229 and LN18 cells which treated with celecoxib in the presence or absence of TMZ, but the combination was more significant which was the respond of the oxidative stress (Figure 5B).

## DISCUSSION

GBM is a serious malignant tumor of the central nervous system, and because of its chemo-resistance has brought great challenges to treatment. TMZ can effectively against human cancers such as melanomas and astrocytoma [27–30]. It was approved by the U.S. Food and Drug Administration (FDA) for use in the treatment of refractory anaplastic astrocytoma in adults in 1999 and newly diagnosed adult GBM patients in 2005 [2]. Regardless of advanced diagnostic modalities and ideal multidisciplinary treatment that includes maximal surgical resection, followed by radiotherapy plus concomitant and maintenance TMZ chemotherapy, almost all patients experience tumor progression with nearly universal mortality. The median survival from initial diagnosis is less than 15 months, with a 2-year survival rate of 26–33% [2, 31], and patients with initial tumor control will inevitably relapse or progress during or after TMZ therapy. Thus, both constitutive and acquired glioma cell resistances to alkylating chemotherapy are major clinical challenges [12].

There were many underlying mechanisms of TMZ resistance. The most characterized culprit of TMZ resistance is the enzyme methylguanine DNA-methyltransferase (MGMT), and MGMT levels have thus been closely associated with clinical outcomes in GBM patients [11, 32]. However, this mechanism is not indisputable. Some studies have confirmed the MGMT is the main factor of TMZ-resistance, other study has objected to that cause of the tumors with the p53 mutated is resistance to TMZ [33, 34]. There is study also show that the cancer stem cells (CSC) is a potential cause of TMZ resistance [35]. Because of so many types of GBM cell line, there is no consensus on which mechanisms affects the resistance of TMZ. All data indicate that TMZ resistance results from a complex cellular response by the GBM cells [36–38]. Although TMZ has been shown to be effective in the treatment of GBM, the TMZ resistance in GBM was common, especially for relapsed acquired chemoresistance GBM, which suggests that developing a new strategy that can increase TMZ sensitivity to GBM is very necessary.

A Phase II clinical trial by the Sidney Kimmel Comprehensive Cancer Center and NCI studied the effectiveness of celecoxib in treating patients who were under treatment with antiepileptic drugs and radiation therapy for newly diagnosed GBM (https://clinicaltrials.gov/show/NCT00068770). Unfortunately, the trial was terminated because an ethical concern. But this given a revelation that the combination TMZ with celecoxib would be a feasible strategy to the GBM. Previous studies have proved that TMZ combined with COX-2 inhibitors can be effective treatment for GBM *in vivo* and *in vitro* but did not explain the molecular mechanism [8, 39]. In our study, it confirms the previous conclusion *in vitro* (Figures 2, 3) and this phenomenon is related to mitochondrial metabolism (Figure 5B).

Shono, T. et al. [40] have reported that high COX-2 expression in tumor cells is associated with clinically more aggressive gliomas and is a strong predictor of poor survival. Most brain tumors, including astrocytoma, glioblastoma, meningioma, medulloblastoma, highly expressed COX-2, and most human malignant glioma cell lines show constitutively elevated levels of COX-2, and an increasing body of evidence from preclinical and clinical studies suggests that elevated COX-2 activity in turn contributes to GBM genesis and progression [41–44]. Our data confirmed that the combination treatment significantly inhibited cell growth than the treatment with TMZ alone which suggests that celecoxib can improve the efficacy of TMZ (Figures 1, 2). A recent study also showed that COX-2 played complex roles in glioma invasion, angiogenesis, immunosuppression, etc. Overexpressed COX-2 contributes to the glioblastoma progression [44]. There were some contradictions about COX-2 inhibitors to GBM invasion, which was probably due to the different type of COX-2 inhibitors. And celecoxib has been reported more potent than those of other selective COX-2 inhibitors or traditional NSAIDs, and which were mediated via the transcriptional inhibition of two essential components of the cell cycle machinery, cyclin A and cyclin B [45]. Our study did not focus on the GBM invasion, follow-up study could be further carried out.

Many studies have explained that the characters of the Warburg effect, tumor hypoxia, genetic mutations, and mitochondrial abnormalities within proliferating cancer cells. Oxidative phosphorylation or electron transport-linked phosphorylation or terminal oxidation) is the metabolic pathway in which cells use enzymes to oxidize nutrients. And this takes place inside mitochondria in eukaryotes. The electron transport chain is a set of enzymes which consisting of complexes I through IV, and the ATP synthase, also called complex V, is the final enzyme in the oxidative phosphorylation pathway. The previous study has shown that the TMZ resistance in Gliomas may due to a mitochondrial adaptive response to TMZ genotoxic stress [7]. Hence, our study focused on the changes of the mitochondrial metabolic enzymes in the LN229 and LN18 cell lines treated with the combination therapy.

A recent study showed that the GBM cancer stem cells (CSC) were primarily responsible for metastatic dissemination, resistance to therapy, and relapse of GBM, LN229 was CSC-enriched GBM cell lines, and easier to resistance to therapy and relapse of GBM [46]. In our data, the LN18 was more effective to the combination therapy than LN229 (Figures 1, 2, and Supplementary Tables 1, 2), possible due to that LN229 was CSC-enriched and more resistance to drug [44]. Collectively, the data indicate that when the LN229 and LN18 treated with 30uM celecoxib, the complex I, II, V were downregulated, in the presence or absence of TMZ (Figure 5B). This suggest that the 30uM celecoxib could acts the ETC and induce the apoptosis (Figure 5C).

Apoptosis as a mechanism of mediated cell death has been widely studied [47], and which is thought to be an anti-cancer molecular mechanism. One of the main apoptotic pathways is intrinsic mitochondrial pathway [48], our study show first found that the celecoxib and combination therapy acts on ETC of mitochondrial and induces the GBM apoptosis by modulating the intrinsic pathway of apoptosis. This result is consistent with previous study that celecoxib could induce apoptosis through the mitochondrial pathway [49]. Jendrossek, V. et al. [50] also concludes that celecoxib induces apoptosis independently from its COX-2 inhibitory action via a mitochondrial apoptosis pathway. The crosstalk between autophagy and apoptosis in cancer is complex, some study point out that the autophagy could suppress apoptosis [51], but others suggest the autophagy could promote apoptosis in certain physiological process of cancer [52, 53]. In our study, the results show that the autophagy increased when the GBM cell lines treated with the combination therapy, and consistent with the latter studies. The autophagy could promote apoptosis in GBM, and this process is due to the celecoxib especially combination therapy.

Our study revealed that the celecoxib significantly enhanced the chemo-sensitization of LN18, this could be demonstrated by the results of cell proliferation, apoptosis, and clone formation. The main reason may be due to the high expression of COX-2 on LN18. When we applied the celecoxib, which is the specific inhibitor of COX-2, the levels of COX-2 were down regulated significantly on LN18. As well as the complex I, II, V, TFAM, NRF2 down regulated significantly (Figure 5). Even the complex III is also downregulated in LN18, but not significantly in LN229 (Figure 5). This finding suggests that the expression of COX-2 might be a new biomarker for chemo sensitization in GBM patients. On the other hand, our study identified the molecular mechanisms of the celecoxib reverses chemo-resistance of GBM to TMZ by inhibiting the OXPHOS, promoting apoptosis and autophagy.

We conclude from these data that the combination therapy (TMZ 250uM+celecoxib 30uM) inhibits cell proliferation, increases apoptosis, and increases the autophagy on LN229 and LN18, which were TMZ resistant GBM cell lines. Celecoxib would reverse chemo-resistance of GBM to TMZ *in vitro*, consistent with previous study [44]. And this study first shows that the potential molecular mechanisms related to mitochondrial metabolism and respiratory chain inhibition. New treatments for GBM are urgently needed and combining celecoxib with temozolomide-based therapy may improve the outcome of these patients.

## MATERIALS AND METHODS

### Chemicals and reagent

TMZ was obtained from Sigma-Aldrich (St. Louis, USA, T2577) and was dissolved in dimethyl sulfoxide (DMSO) Sigma-Aldrich (St. Louis, USA, D2650) to obtain a final concentration of 100 mM. Celecoxib was obtained from Sigma-Aldrich (St. Louis, USA, PZ0008) which was also dissolved in DMSO to obtain a final concentration of 100 mM. DMSO was kept below 0.35 % in all cell culture experiments. MTS was obtained from Promega (Fitchburg, WI, USA, G3582). Crystal violet was obtained from Sigma-Aldrich (St. Louis, USA, AC405830250). Hoechst 33342/Propidium Iodide was obtained from Thermo Fisher Scientific (ShangHai, China, V13244). PSVue 643 was obtained from Molecular Targeting Technologies (West Chester PA, USA, P-1006).

### Cell lines and culture conditions

Human GBM cells, LN18 and LN229, were purchased from American Type Culture Collection (ATCC) and cultured following their culture procedures. The cells were cultured with Dulbecco’s minimum essential medium (DMEM) containing 10% fetal bovine serum (FBS) and cultured at 37 ° C and in 5% CO2.

### Cell cytotoxicity / proliferation assay with MTS

Cell cytotoxicity / Proliferation was performed as following. Briefly, Plate cells at appropriate density (5000 cells/well) in 100uL media (3 wells per condition). And make sure to have three wells with only media at the bottom, add 50uL diH20 to outside wells to control for edge effects. The cells adhere to plate after incubating for 24hrs, then add 20uL CellTiter 96® AQueous One Solution Reagent to Day 0 wells (want 5 parts media:1 part MTS reagent; ex. 100ml media:20ul MTS reagent), then absorbance was measured at 490nm after 1hr.

### Crystal violet staining assay

Crystal violet staining was performed as following. Briefly, Plate cells at appropriate density (2000 cells/well) with media contains TMZ, cultured for 2 weeks. Then take off media, to making sure not to touch top to bottom of well. Wash wells with 1X cold PBS and add 0.5ml 0.25% crystal violet staining solution to each well, then incubate at room temperature for 30 minutes. Take off crystal violet and dispense into waste bottle. Keep plate submerged and keep water running until water in tub is no longer purple. Place plates upside down and leave to dry overnight.

### Fluorescence-activated cell sorting (FACS) assay

The cell-apoptosis was determined by flow cytometry. The cells were treated with TMZ, celecoxib, combination for 72h, then harvested. They were then pelleted, washed with PBS and resuspended in propidium iodide (PI) solution (50 μg/mL PI, 0.5 mg/mL RNase staining buffer) and PSVue 643 for 15 min in the dark. Data were collected and analyzed using the Cellfit program with a FACS can flow cytometer (FACS Canto II Cytometer: BD Biosciences, San Jose, CA).

### Western blot analysis

Cells were washed twice with PBS and lysed with a lysis buffer (Trizma base 50mM, sucrose 0.25mM, EDTA 5mM and triton X-100 0.5%, pH 7.4). Protein concentration was determined using Bradford Reagent (Bio-Rad) after sonication. Thus, 25μg of protein was electrophoretically separated by 12% SDS-PAGE and transferred to nitrocellulose membranes. These membranes were blocked for 60 min at room temperature in 5% (w/v) milk powder in TBS containing 0.1% Tween 20, co-incubated overnight at 4° C with the primary antibodies (TFAM 1:1000, LC3B 1:1000, Cell Signaling Technology; ATP5A1 1:500, NDUFB8 1:500, SDHA 1:500, UQCRC1 1:500, NRF2 1:600, COX2 1:600, Proteintech; β-actin 1:10000, Cell Signaling Technology), washed three times with 0.1% Tween 20 in TBS, and incubated for 1 h with a horseradish peroxidase-conjugated (HRP) goat anti-Rabbit secondary antibody 1:5000 (Cell Signaling Technology). Proteins were visualized using the ECL system (Amersham Biosciences, USA) in the Bio-Rad equipment. Further analysis, as well as image processing and quantification of the bands, was performed using the program Image lab. Expression was normalized relative to the β-actin level.

### Fluorescence microscopy assay

Hoechst 33342 and PI staining were performed as the protocol. Briefly, the GBM cells of control, TMZ, celecoxib, and combination groups were prepared in the 12 well-plates after treated with inhibitors 36h, and then the Hoechst33342 and PI solution by diluting the Hoechst stock solution 1:2000 in PBS and the same dilution of the PI in the same PBS. Remove the media, add sufficient staining solution 300ul to cover the cells, and incubate for 5-10mins in the dark room. Finally, the images were collected by using EVOS Cell Imaging Systems f1 (Thermo Fisher Scientific, USA).

### Statistical analysis

Experimental data is presented as the mean ± standard deviation of three experimental repeats. GraphPad Prism 8.0 (GraphPad Software Corp., USA) was used to perform a two-way ANOVA analysis of variance with the least significant difference post hoc test. P≤0.05 was considered to indicate a statistically significant difference.

## Supplementary Material

Supplementary Table 1

Supplementary Table 2

Supplementary Tables 3 and 4
