# Supplementary material for: Celecoxib reverses the glioblastoma chemo-resistance to temozolomide through mitochondrial metabolism
Source: Aging (Albany NY). 2021 Sep 8;13(17):21268–82. doi: 10.18632/aging.203443 (PMC8457578; doi:10.18632/aging.203443)
Supplement: Supplementary Tables 3 and 4 [file aging-13-203443-s003.pdf]

SUPPLEMENTARY TABLES

Supplementary Table 3. Statistical analysis of clone formation experiment.

2wayANOVA									
**Multiple comparisons**									
**1**	Within each row, compare columns (simple effects within rows)								
**2**									
**3**	Number of families	2							
**4**	Number of comparisons per family	6							
**5**	Alpha	0.05							
**6**									
**7**	Tukey's multiple comparisons test	Mean Diff.	95.00% Cl of diff.	Significant?	Summary	Adjusted P Value			
**8**									
**9**	LN229								
**10**	DMSO vs. TMZ 250uM	0.07728	-0.1011 to 0.2557	No	ns	0.6475			
**11**	DMSO vs. Celecoxib 30uM	0.3435	0.1651 to 0.5219	Yes	****	<0.0001			
**12**	DMSO vs. Celecoxib 30uM+TMZ 250uM	0.6027	0.4243 to 0.7811	Yes	****	<0.0001			
**13**	TMZ 250uM VS. Celecoxib 30uM	0.2663	0.08785 to 0.4447	Yes	**	0.0017			
**14**	TMZ 250uM VS. Celecoxib 30uM+TMZ 250uM	0.5255	0.3471 to 0.7039	Yes	****	<0.0001			
**15**	Celecoxib 30uM vs. Celecoxib 30uM+TMZ 250uM	0.2592	0.08079 to 0.4376	Yes	**	0.0023			
**16**									
**17**	LN18								
**18**	DMSO vs. TMZ 250uM	0.03566	-0.1428 to 0.2141	No	ns	0.9482			
**19**	DMSO vs. Celecoxib 30uM	0.3534	0.175 to 0.5318	Yes	****	<0.0001			
**20**	DMSO vs. Celecoxib 30uM+TMZ 250uM	0.6174	0.439 to 0.7958	Yes	****	<0.0001			
**21**	TMZ 250uM vs. Celecoxib 30uM	0.3178	0.1394 to 0.4962	Yes	***	0.0002			
**22**	TMZ 250uM vs. Celecoxib 30uM+TMZ 250uM	0.5818	0.4034 to 0.7602	Yes	****	<0.0001			
**23**	Celecoxib 30uM vs. Celecoxib 30uM+TMZ 250uM	0.264	0.08559 to 0.4424	Yes	**	0.0018			
**24**									
**25**									
**26**	Test details	Mean 1	Mean 2	Mean Diff.	SE of diff.	N1	N2	q	DF
**27**									
**28**	LN229								
**29**	DMSO vs. TMZ 250uM	1.015	0.9378	0.07728	0.06585	5	5	1.66	32
**30**	DMSO vs. Celecoxib 30uM	1.015	0.6715	0.3435	0.06585	5	5	7.378	32
**31**	DMSO vs. Celecoxib 30uM+TMZ 250uM	1.015	0.4123	0.6027	0.06585	5	5	12.94	32
**32**	TMZ 250uM vs. Celecoxib 30uM	0.9378	0.6715	0.2663	0.06585	5	5	5.718	32
**33**	TMZ 250uM vs. Celecoxib 30uM+TMZ 250uM	0.9378	0.4123	0.5255	0.06585	5	5	11.29	32
**2wayANOVA**									
**Multiple comparisons**									
**34**	Celecoxib 30uM vs. Celecoxib 30uM+TMZ 250uM	0.6715	0.4123	0.2592	0.06585	5	5	5.567	32
**35**									
**36**	LN18								

**37**	DMSO vs. TMZ 250uM	1	0.9643	0.03566	0.06585	5	5	0.7658	32
**38**	DMSO vs. Celecoxib 30uM	1	0.6466	0.3534	0.06585	5	5	7.59	32
**39**	DMSO vs. Celecoxib 30uM+TMZ 250uM	1	0.3826	0.6174	0.06585	5	5	13.26	32
**40**	TMZ 250uM vs. Celecoxib 30uM	0.9643	0.6466	0.3178	0.06585	5	5	6.824	32
**41**	TMZ 250uM vs. Celecoxib 30uM+TMZ 250uM	0.9643	0.3826	0.5818	0.06585	5	5	12.49	32
**42**	Celecoxib 30uM vs. Celecoxib 30uM+TMZ 250uM	0.6466	0.3826	0.264	0.06585	5	5	5.67	32

Supplementary Table 4. Statistical analysis of apoptosis experiment.

**2wayANOVA Multiple comparisons**									
**1**	Within each row, compare columns (simple effects within rows)								
**2**									
**3**	Number of families	2							
**4**	Number of comparisons per family	6							
**5**	Alpha	0.05							
**6**									
**7**	Tukey's multiple comparisons test	Mean Diff.	95.00% Cl of diff.	Significant?	Summary	Adjusted P Value			
**8**									
**9**	LN229								
**10**	DMSO vs. TMZ 250uM	-0.41	-2.241 to 1.421	No	ns	0.9292			
**11**	DMSO vs. Celecoxib 30uM	-2.28	-4.111 to -0.4495	Yes	*	0.0100			
**12**	DMSO vs. Celecoxib 30uM+TMZ 250uM	-5.59	-7.421 to -3.759	Yes	****	<0.0001			
**13**	TMZ 250uM VS. Celecoxib 30uM	-1.87	-3.701 to -0.0395	Yes	*	0.0438			
**14**	TMZ 250uM VS. Celecoxib 30uM+TMZ 250uM	-5.18	-7.011 to -3.349	Yes	****	<0.0001			
**15**	Celecoxib 30uM vs. Celecoxib 30uM+TMZ 250uM	-3.31	-5.141 to -1.479	Yes	***	0.0002			
**16**									
**17**	LN18								
**18**	DMSO vs. TMZ 250uM	-1.65	-3.481 to 0.1805	No	ns	0.0893			
**19**	DMSO vs. Celecoxib 30uM	-8.23	-10.06 to -6.399	Yes	****	<0.0001			
**20**	DMSO vs. Celecoxib 30uM+TMZ 250uM	-23.4	-25.23 to -21.57	Yes	****	<0.0001			
**21**	TMZ 250uM vs. Celecoxib 30uM	-6.58	-8.411 to -4.749	Yes	****	<0.0001			
**22**	TMZ 250uM vs. Celecoxib 30uM+TMZ 250uM	-21.75	-23.58 to -19.92	Yes	****	<0.0001			
**23**	Celecoxib 30uM vs. Celecoxib 30uM+TMZ 250uM	-15.17	-17 to -13.34	Yes	****	<0.0001			
**24**									
**25**									
**26**	Test details	Mean 1	Mean 2	Mean Diff.	SE of diff.	N1	N2	q	DF
**27**									
**28**	LN229								
**29**	DMSO vs. TMZ 250uM	4.56	4.97	-0.41	0.6756	5	5	0.8582	32
**30**	DMSO vs. Celecoxib 30uM	4.56	6.84	-2.28	0.6756	5	5	4.773	32
**31**	DMSO vs. Celecoxib 30uM+TMZ 250uM	4.56	10.15	-5.59	0.6756	5	5	11.7	32
**32**	TMZ 250uM vs. Celecoxib 30uM	4.97	6.84	-1.87	0.6756	5	5	3.914	32
**33**	TMZ 250uM vs. Celecoxib 30uM+TMZ 250uM	4.97	10.15	-5.18	0.6756	5	5	10.84	32
**2wayANOVA Multiple comparisons**									
**34**	Celecoxib 30uM vs. Celecoxib 30uM+TMZ 250uM	6.84	10.15	-3.31	0.6756	5	5	6.929	32
**35**									

**36**	LN18								
**37**	DMSO vs. TMZ 250uM	6.44	8.09	-1.65	0.6756	5	5	3.454	32
**38**	DMSO vs. Celecoxib 30uM	6.44	14.67	-8.23	0.6756	5	5	17.23	32
**39**	DMSO vs. Celecoxib 30uM+TMZ 250uM	6.44	29.84	-23.4	0.6756	5	5	48.98	32
**40**	TMZ 250uM vs. Celecoxib 30uM	8.09	14.67	-6.58	0.6756	5	5	13.77	32
**41**	TMZ 250uM vs. Celecoxib 30uM+TMZ 250uM	8.09	29.84	-21.75	0.6756	5	5	45.53	32
**42**	Celecoxib 30uM vs. Celecoxib 30uM+TMZ 250uM	14.67	29.84	-15.17	0.6756	5	5	31.75	32
